# Mechanical Properties and Fatigue Failure of Thermally and Thermochemically Treated C60 Steel

**DOI:** 10.3390/ma19153239

**Published:** 2026-07-30

**Authors:** Iuliana Tudorache (Nistor), Cornel Samoila, Doru Ursutiu

**Affiliations:** 1 Faculty of Material Science and Engineering, Transylvania University of Brasov, Bdul. Eroilor Nr. 29, 500036 Brasov, Romania; csam@unitbv.ro; 2 Technical Science Academy of Romania, Bd. Dacia Nr. 26, Sector 1, 010413 Bucharest, Romania; 3 Faculty of Electrical Engineering and Computer Science, Transylvania University of Brasov, Bdul. Eroilor Nr. 29, 500036 Brasov, Romania; ursutiu@unitbv.ro; 4 Academy of Romanian Scientists, Str. Ilfov Nr. 3, Sector 5, 030167 Bucharest, Romania

**Keywords:** heat treatment, oxidation, fatigue crack monitoring, C60 steel

## Abstract

The present study is situated within the broader research context of optimizing the use of C60 steel in industrial applications, with a particular emphasis on enhancing durability and fatigue resistance, two critical factors in the field. C60 steel is renowned for its exceptional combination of strength and hardness. However, a critical evaluation is necessary to ascertain the impact of thermal and thermochemical treatments on its performance under fatigue conditions. An investigation was conducted into the crack formation process and the early stages of fatigue in C60 steel. The effects of heat treatment (hardening and tempering) were compared with those of thermochemical treatments (oxidation) on the steel’s microstructure. Furthermore, the performance of C60 steel under fatigue conditions was evaluated based on the applied treatments. The insights gained from this research can optimize the use of this steel in industries requiring high resistance and durability. The study’s methodology encompassed the execution of fatigue tests on a four-point bending machine, a procedure that was meticulously employed to ascertain the onset of microcracking. The C60 steel samples were processed in accordance with SR ISO 1099:2017, entitled “Fatigue testing. Axial load method”. To ensure consistency and comparability of results, the samples were fabricated from the same material charge and machined under identical conditions. A total of 26 samples were utilized, with 13 samples allocated to each treatment type: heat treatment by hardening, tempering, and thermochemical treatment by oxidation. To ensure comparability and scientific interpretability of the results, an identical applied force level was utilized for both treatment conditions. The frequency changes were monitored to evaluate the behavior of the materials under repeated stresses. Finally, the frequency changes were correlated with the number of cycles to identify when microcracks appeared and their evolution. The primary findings of this study indicate substantial disparities between the longevity of thermally and thermochemically modified specimens and the onset of microcrack formation in the material.

## 1. Introduction

The fatigue behavior of metallic materials has been a central topic in the field of fracture mechanics since the seminal works of Griffith [1], Irwin [2], and Paris–Erdogan [3]. Contemporary models developed by Suresh [4] and Anderson [5] demonstrate that the initiation of cracks is significantly influenced by microstructure, surface condition, and the distribution of local stresses. This underscores the necessity of fatigue analysis in the design of components subjected to cyclical loading. Surface defects, roughness, and nonmetallic inclusions have been shown to significantly reduce fatigue strength (see [6,7]). In the VHCF regime, crack initiation may shift from the surface to the interior of the material (see [8]).

The following investigation will explore the mechanisms of crack initiation. In the VHCF regime, the mechanisms that initiate cracks exhibit distinct characteristics. Mughrabi [9] demonstrated that cyclic strain localization governs the onset of cracking. For medium-carbon steels, Koster et al. [10] highlighted the influence of multiphase microstructures and the stability of retained phases. 

According to Reifsneider [11], who studied the long-term evolution of composite materials and structures subjected to time-varying mechanical, thermal, and chemical influences, material fatigue is a permanent, localized, and progressive structural change that occurs in materials subjected to alternating stresses, ultimately leading to cracking or failure after a sufficient number of cycles.

Several authors have developed semi-analytical models for estimating the number of cycles required for crack initiation. Cheng [12] proposed a predictive micromechanical approach whose accuracy was confirmed through experimental validation.

The study presented in [13] demonstrates that cracks originate within the surface oxide layers, which are inherently brittle and subjected to the highest tensile stresses, leading to early fatigue crack initiation. Furthermore, oxygen ingress through these layers accelerates crack propagation.

Zhang, Li, and Chen [14] investigated how high-temperature oxidation affects fatigue crack initiation in hot-work tool steels. Their results show that the formation of oxide layers alters the surface condition and promotes earlier crack initiation under cyclic loading, thereby reducing the fatigue resistance of the material.

The paper [15] introduces a multi-task deep learning framework, MT-CrackNet, designed for the automated detection and quantification of in situ fatigue microcracks, enabling real-time monitoring and measurement during fatigue testing.

The internal structure of the material, which includes phase transformations, grain-boundary sliding, porosity, and the distribution of inclusions, has been shown to control its response to cyclic loading. As indicated by the seminal work of ref. [16], the presence of internal defects may result in the formation of fish-eye cracks within the VHCF. Furthermore, the spatial distribution of inclusions exerts a significant influence on the early stages of fatigue damage, as evidenced by the findings reported in [17].

### 1.1. Crack Propagation and Predictive Approaches

In ref. [18], the authors examine crack initiation and growth by linking microstructural characteristics to fatigue-damage mechanisms. Crack propagation is controlled by the stress level, the maximum stress introduced into the material, and occurs through repeated deformation and failure near the crack tip. With increasing crack length, the deterioration process is accelerated. There are situations where, due to the low-stress level, the crack propagation ceases. The fatigue life of a structure is provided by the number of stress cycles after which failure occurs. The current method of estimating the fatigue life of structures is to calculate the equivalent stress amplitude from measured stress data.

As demonstrated in the following reference, crack propagation is governed by the applied stress level and evolves through repeated deformation and localized failure near the crack tip. The fatigue life of a material is determined by the number of cycles it can withstand before reaching failure. Modern prediction methods rely on the equivalent stress amplitude [19]. In resonance-based fatigue testing, damage is commonly monitored through reductions in natural frequency [20,21,22], which correlate with stiffness loss as cracks initiate and grow. A multitude of studies have demonstrated that frequency-based monitoring provides a reliable indicator of crack initiation and propagation in vibrating specimens [23,24,25,26], thus establishing it as a widely used criterion for defining failure in high-frequency fatigue experiments. To complement frequency-based monitoring, Digital Image Correlation (DIC) has been widely adopted to quantify crack-tip deformation, evaluate stress intensity factors, and identify crack propagation laws with high spatial resolution [27,28,29,30,31,32]. However, the majority of DIC applications have centered on low-cycle fatigue.

The impact of microstructural evolution, the conditions under which heat treatment is applied, and the phenomenon of oxidation-assisted damage on the initiation of fatigue cracks has been demonstrated across a broad spectrum of alloys and steels [33,34,35,36,37,38].

### 1.2. A Lack of Knowledge and Motivation

Despite the extensive research conducted on the subject of fatigue behavior, the early stages of microcrack initiation in medium-carbon steels—particularly under combined thermal and thermochemical oxidation—remain insufficiently understood. For C60 steel, a material frequently utilized in industrial applications, there is a paucity of data concerning microcrack initiation under high-frequency cyclic loading conditions.

The objective of the present study is to investigate the mechanisms of microcrack initiation in C60 steel that has been subjected to quenching, tempering, and thermochemical oxidation. The present study utilizes a meticulous approach to elucidate the influence of surface oxidation and microstructural modifications on fatigue behavior. This objective is achieved by meticulously monitoring frequency changes during four-point bending fatigue tests and conducting a comparative analysis of the onset and evolution of microcracks between the two treatment conditions.

## 2. Materials and Methods

### 2.1. Materials

**Figure 1:**
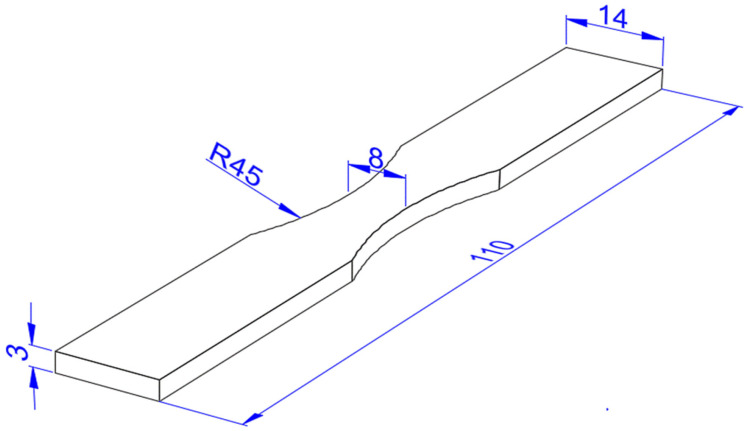
Geometry and dimensions of the fatigue test Sample, machined according to SR ISO 1099:2017.

In this study, the properties of C60 steel were examined under two distinct material conditions: heat-treated by quenching and tempering (the material was supplied by Zelos Zerspanung, Bessenbach, Germany) and thermochemically treated by gas oxidation, a process that was carried out at Härterei Reese Brackenheim, Brackenheim, Germany. The material was delivered as a flat bar with dimensions of 110 × 14 × 3 mm, and the samples were machined in accordance with SR ISO 1099:2017 [39] (Figure 1).

In order to ensure consistency and comparability between the two material conditions, it was imperative that all samples be manufactured from the same material batch and machined under identical technological parameters.

The fatigue testing procedure was executed using a four-point bending configuration. This configuration was meticulously selected to facilitate the precise detection of microcrack formation and to systematically monitor their evolution under cyclic loading.

The chemical composition was determined using high-resolution spark spectrometry, employing the SPECTROMAXx spectrometer (SPECTRO Analytical Instruments GmbH, Kleve, Germany) from Liebherr Deggendorf Laboratory (Deggendorf, Germany). To this end, nine measurements were conducted to obtain precise and relevant values, which are essential for the detailed characterization of the material.

The chemical composition of the alloy is presented in Table 1.

### 2.2. Methods

#### 2.2.1. Fatigue Testing

In order to execute experiments designed to induce fatigue and subsequently analyze the material’s response to repetitive loading, a meticulously formulated testing protocol has been devised in close collaboration with numerous corporate entities and research institutions.

To ensure clear traceability, the samples were marked via laser engraving, facilitating their identification throughout the testing process.

**Figure 2:**
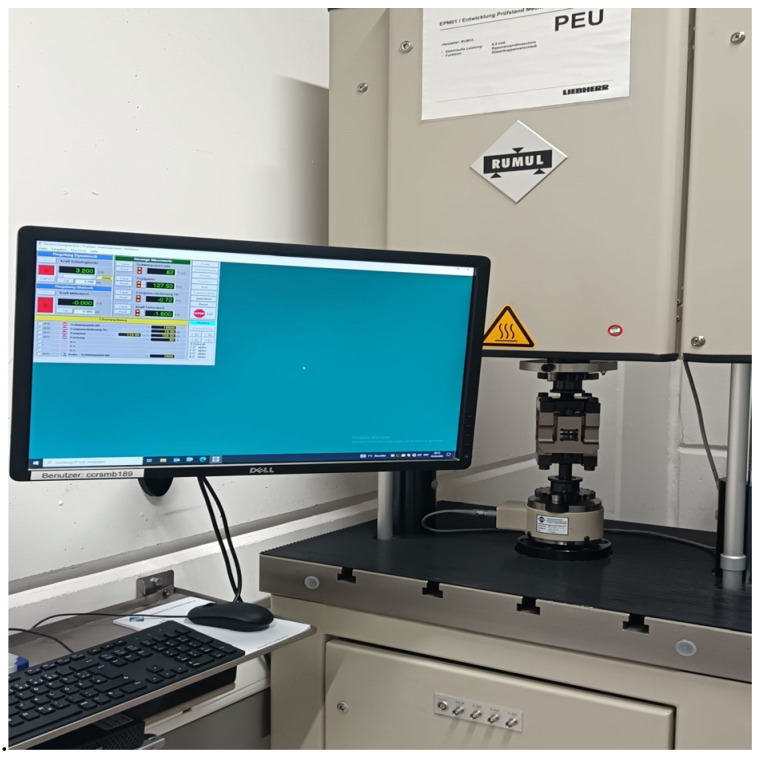
Four-point bending fatigue testing machine (MIKROTRON, 100 Nm/20 kN) used for high-frequency cyclic loading.

For the fatigue experiments, a total of 26 samples were prepared, of which 13 were tested in the quenched and tempered condition and 13 after thermochemical oxidation. The testing of all samples was conducted at the manufacturing facility. Liebherr Deggendorf utilizes a MIKROTRON four-point bending machine (RUMUL, Russenberger Prüfmaschinen, Neuhausen am Rheinfall, Switzerland) (100 Nm/20 kN) that has been specifically designed for high frequency fatigue testing (see Figure 2). The machine is capable of operating at frequencies up to 140 Hz; in this study, all tests were performed at a nominal frequency of 127 ± 10 Hz.

**Figure 3:**
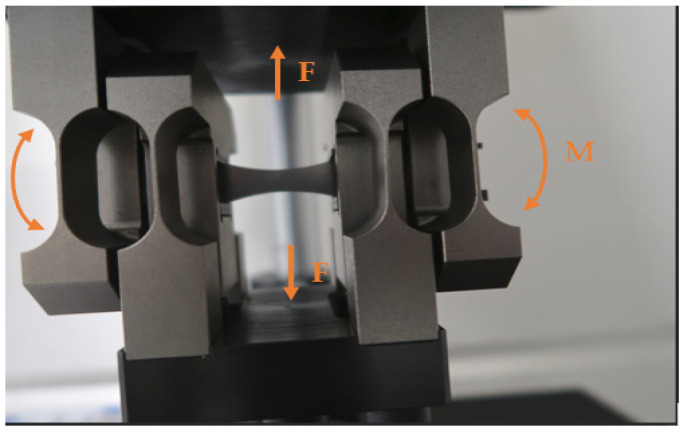
Sample wedge grip.

As illustrated in Figure 3, the gripping system plays a pivotal role in ensuring the stability of the sample during fatigue tests. This system is designed to facilitate proper force transmission and prevent unwanted movements during load-cycle reversal, thereby maintaining the integrity of the sample throughout the testing process.

- In the four-point bending test, the sample is supported at pairs of two points. The utilization of counter-nuts is imperative in the elimination of gaps, given the execution of tests in an alternating cycle.
- The gripping areas of the testing machine wedge grips are machined by milling, a process which ensures the alignment of the wedge grip and test sample with the direction of the loading force.
- The forces (F) are applied vertically at two symmetrical points, indicating a bending-type loading.
- The bending moments (M) are generated between the points of force application and the support points, as illustrated by curved arrows.
- The tightening of the testing machine wedge grip is accomplished through the utilization of a hydraulic mechanism, thereby ensuring the secure clamping of the sample and averting the potential for slippage or loss of the specimen during the reversal of force. In light of these conditions, it can be posited that the sample axis is correctly aligned with the direction of the loading force and that all gripping elements are adequately tightened, thereby eliminating gaps between the sample’s clamping components.

The fatigue tests were executed under a constant loading regime to ensure strictly controlled and comparable conditions between the thermally treated and the thermo-chemically oxidized specimens. The decision to employ a single loading level was made with the objective of facilitating a clear observation of the microcrack initiation mechanisms, without the confounding influence of varying stress amplitudes.

The present article evaluates tests conducted under stress control exclusively within the field of temporary fatigue resistance. In this scenario, the material exhibits sufficient resistance to withstand repetitive cyclic loading for a specified number of cycles prior to the onset of crack formation. The present method is distinct from the concept of unlimited fatigue resistance, wherein the material exhibits remarkable resilience, capable of withstanding a substantial number of cycles without manifesting any significant deterioration.

The findings of this investigation are pivotal in elucidating the mechanisms underlying material degradation. A decrease in frequency can offer valuable insights into the formation and propagation of cracks, as well as the impact of such decreases on material properties. Mechanical reasoning is predicated on the observation of the behavior of materials under conditions of repetitive stress. The reduction in testing frequency is employed to identify the initiation and failure of cracks in the material. A minor decline in frequency is indicative of the onset of deterioration, while a substantial decrease signifies material failure. The aforementioned variations have been demonstrated to be correlated with stiffness degradation and crack length growth, thereby enabling precise monitoring of deterioration. In this context, for all tests conducted, a reduction in testing frequency by 0.02 hertz (Hz) was established as the crack initiation criterion, while a reduction of 1.2 hertz (Hz) was set as the failure criterion. This established critical thresholds for evaluating the system’s behavior under stress conditions. Furthermore, the relationship between the decline in frequency and the deterioration of stiffness was substantiated through in situ measurements. The experimental protocol entailed the continuous monitoring of frequency during fatigue tests, complemented by the direct observation of cracks through imaging techniques such as computed tomography.

#### 2.2.2. Thermochemical Oxidation

Subsequent to a meticulous cleaning process, the metal components were introduced into the furnace and meticulously arranged to ensure they did not overlap. The oxidation process was conducted within a rotary retort furnace, where a controlled nitrogen-based atmosphere and water vapor injection were employed. To mitigate thermal stresses in the oxide layer, the samples were gradually cooled within the furnace.

**Figure 4:**
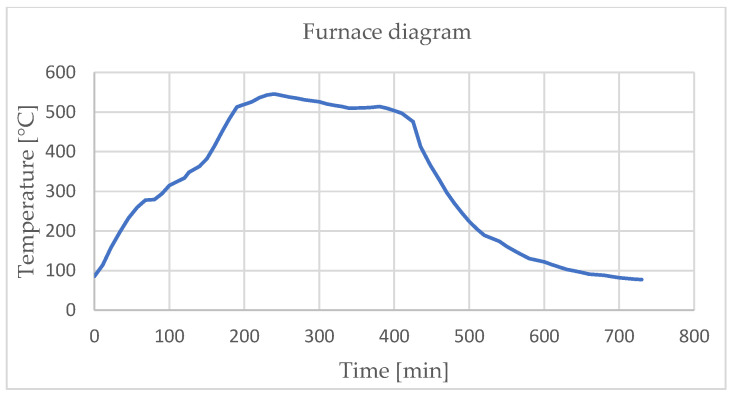
Cyclogram of the oxidation process.

The thermochemical oxidation process has a total duration of approximately 11 h, as demonstrated by the complete thermal cycle in Figure 4.

The accompanying diagram illustrates the furnace temperature changes throughout the treatment process. Over the initial 3.3 h, the temperature undergoes a gradual increase from approximately 100 °C to around 540 °C. This stage is referred to as the “heating stage”, in which the material is elevated to the desired temperature. The oxidation process is initiated during the subsequent 3.3-h period, during which the temperature is maintained within the range of 520 to 540 °C. This allows for the formation and stabilization of a protective oxide layer. Subsequent to this stage, the furnace undergoes a gradual and regulated cooling process, resulting in a temperature reduction to approximately 100 °C by the conclusion of the 11-h cycle.

During this phase, the material’s upper layer is subjected to an oxidizing environment, thereby forming a protective oxide layer. Nitrogen (N_2_) was utilized as the carrier gas to regulate the atmosphere. During the oxidation process, water was introduced into the furnace with the objective of promoting the formation of the oxide layer. Subsequent to oxidation, the samples were gradually cooled to mitigate stresses within the oxide layer.

**Figure 5:**
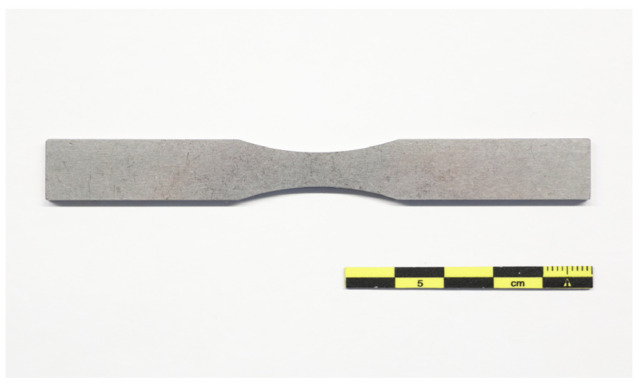
Sample before oxidation.

**Figure 6:**
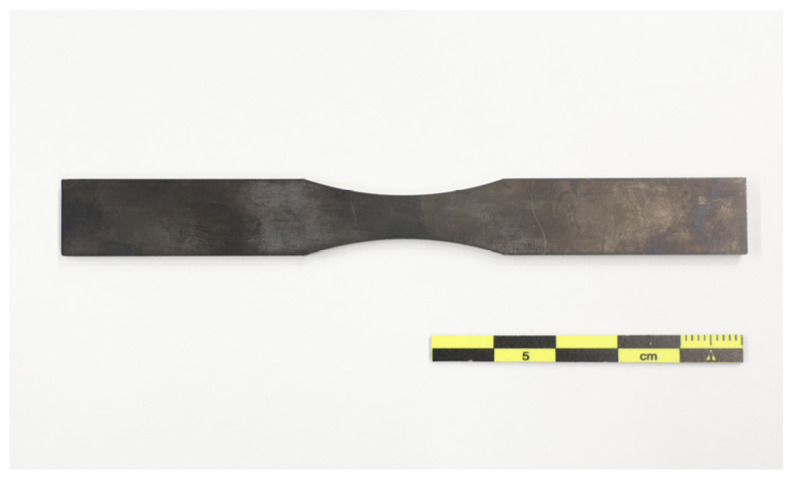
Sample after oxidation.

Following the thermochemical treatment, the samples exhibited a black tint over the entire surface, thereby highlighting the uniformity and efficiency of the oxidation process (Figure 5 and Figure 6).

## 3. Results and Discussion

### 3.1. Hardness Measurements for the Oxidized Sample

The surface of the part was evaluated using a microhardness test (HV 0.05 kgf) to determine whether the thermochemical oxidation process influenced the hardness characteristics. The core of the part was subjected to testing using the Vickers method HV 30 with the KB30S device from Härterei Reese Brackenheim (Brackenheim, Germany). The obtained results were converted into MPa in accordance with DIN EN ISO 18265 [41], Table A1, to ensure a more precise and comparable interpretation.

As illustrated in Table 2, the specimen exhibits a surface hardness of 452 HV 0.05, indicative of a mechanical strength (Rm) of 1453 MPa. Additionally, the core hardness is determined to be 294 HV 30, corresponding to a mechanical strength (Rm) of 920 MPa. These measurements have been converted in accordance with DIN EN ISO 18265, Table A1 (average value of three measurements).

### 3.2. Hardness Measurements for the Hardened and Tempered Sample

The hardness of the part was evaluated through two distinct methods: the Brinell method HBW 2.5/187.5, which utilized the KB 250BVRZ device from Härterei Reese Brackenheim, and the Vickers method HV30, employing the KB30S device. The obtained results were converted into MPa according to DIN EN ISO 18265, Table A1 units, for a more precise and comparable interpretation.

As illustrated in Table 3, the specimen exhibits a surface hardness of 415 HBW, indicative of a mechanical strength (Rm) of 1312 MPa. This is complemented by a core hardness of 424 HV 30, corresponding to an Rm of 1322 MPa, as determined in accordance with DIN EN ISO 18265, Table A1 (average value of three measurements).

### 3.3. Evolution of the Microstructure of the Oxidized Sample

A cross-section of the oxidized sample was cut using a QATM Brillant 220 machine with a SiC wheel at a low feed rate (0.05 mm/s) to avoid residual stress. The sectioned sample was then subjected to hot mounting using the QATM OPAL x-Press system, followed by grinding from P180 to P1800 and polishing with Mono diamond suspension. The microstructural analysis was conducted using an Axio Imager 2 light microscope (Carl Zeiss, Oberkochen, Germany) with ×1000 magnification. In order to avert the occurrence of oxide-layer exfoliation during the preparatory phase, the samples were enveloped in aluminum foil. The metallographic procedure entailed a series of steps, including washing, degreasing with alcohol, drying, and etching with 3% Nital, followed by rinsing and rapid hot-air drying. The microstructure was examined in both the edge and the core to verify the uniformity of the applied heat treatment.

**Figure 7:**
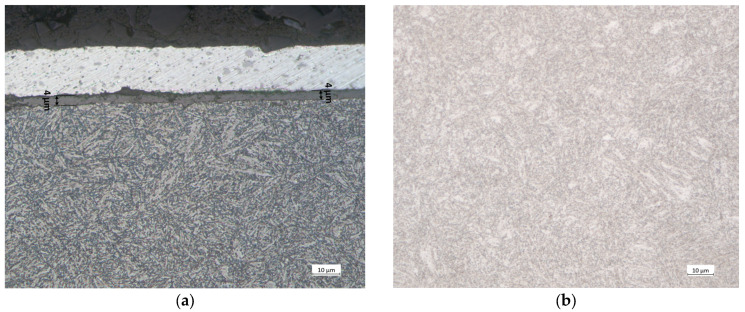
(**a**) Surface microstructure of C60 steel subjected to thermochemical oxidation treatment with an oxide layer of 4 µm. (**b**) The core microstructure of the part, consisting of an intermediate bainitic structure.

Figure 7a illustrates the microstructure of the sample that was thermochemically treated by oxidation. It is evident in the figure that an oxide layer of approximately 4 µm in thickness has formed on the surface of the part. As illustrated in Figure 7b, the core of the part was maintained at a temperature of 500 °C for approximately 2.5 h, followed by a gradual cooling process. This resulted in the observation of an intermediate bainitic structure, which can be described as a mechanical mixture of supersaturated ferrite with carbon and carbides that have not yet reached the stage of cementite.

**Figure 8:**
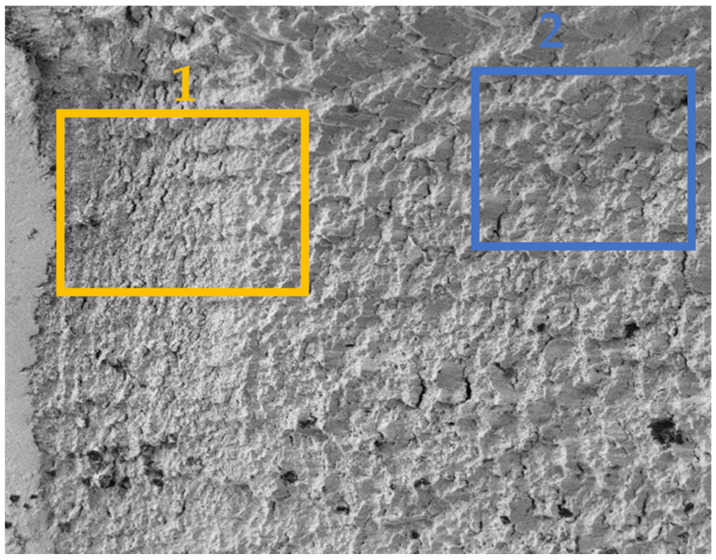
SEM-BSE image of the oxidized layer with the EDS acquisition areas (Spectrum 1—oxidized layer; Spectrum 2—base material).

The oxidized layer was analyzed by scanning electron microscopy (SEM) in both secondary electron (SE) and backscattered electron (BSE) modes. The latter provided superior contrast between the base material and the surface layer (see Figure 8). In this region, two EDS spectra were acquired: Spectrum 1 corresponds to the oxidized layer, and Spectrum 2 corresponds to the base material. Spectrum 1 displays significant nitrogen (N) and oxygen (O) peaks, elements that are not present in the base material. This observation confirms the formation of a distinct oxidized layer.

**Figure 9:**
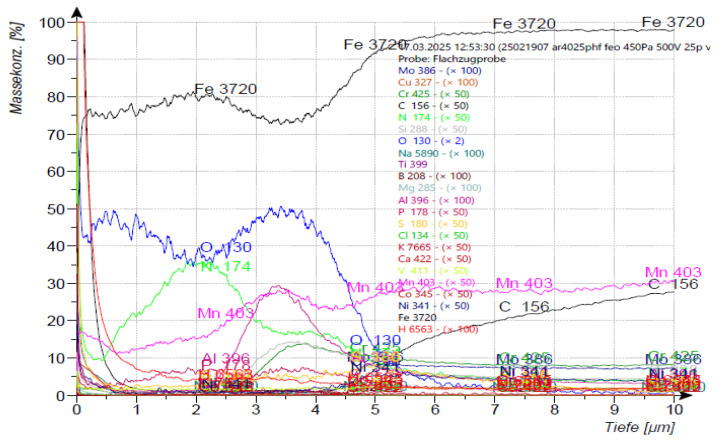
Profiling GDOS analysis.

In order to establish a correlation between the SEM/EDS observations and the depth-dependent elemental distribution, a GDOES analysis was conducted (see Figure 9). A preliminary analysis of the material’s surface layer reveals the presence of oxygen-affine elements, including aluminum, manganese, and silicon, in specific localized areas. The presence of these accumulations was determined through the implementation of GDOES analyses. The experimental findings indicated the presence of elevated concentrations of these elements at depths of a few micrometers, thereby suggesting that diffusion during the thermochemical treatment played a significant role in the formation of the surface layer. At a depth of approximately 5 µm, a significant deposition of elements such as Al, Mn, N, and O can be observed. These elements play crucial roles in the oxidized layer: aluminum (Al) enhances corrosion resistance, manganese (Mn) improves hardness and wear resistance, nitrogen (N) is present in the surface layer, suggesting a possible interaction with the base material during the treatment, without confirming the formation of nitride compounds, and oxygen (O) is essential for the formation of the protective oxide layer. Beyond 5 µm, the material reverts to its original composition, indicating that the oxidized layer is confined to the first few micrometers. As depth is increased, the concentrations of the elements aluminum (Al), manganese (Mn), nitrogen (N), and oxygen (O) decrease exponentially. Aluminum (Al) demonstrates the fastest decrease, indicating a higher presence at the surface. Manganese (Mn) decreases more slowly compared to Al. Nitrogen (N) decreases even more slowly, suggesting deeper diffusion. Oxygen (O) decreases the fastest, indicating a superficial oxide layer.

### 3.4. Evolution of the Microstructure for Hardened and Tempered Sample

**Figure 10:**
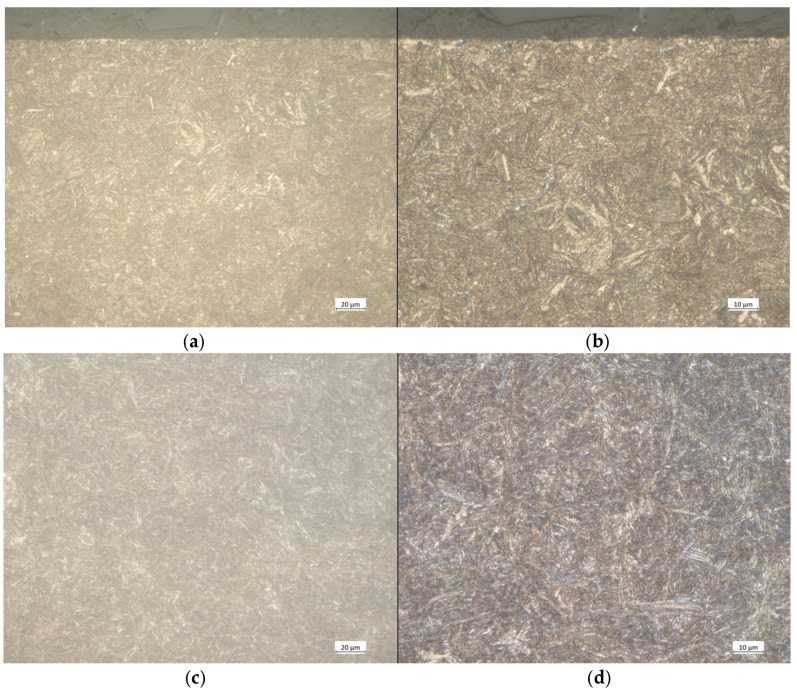
Microstructure of C60 steel after hardening and tempering: (**a**) edge region at 500× magnification; (**b**) edge region at 1000× magnification; (**c**) core region at 500× magnification; (**d**) core region at 1000× magnification.

Metallographic analysis of the hardened and tempered material was conducted, revealing a sorbitic structure composed of a ferritic matrix with uniformly distributed iron carbides (cementite) (see Figure 10). The microstructure exhibited uniformity across the entire cross section, with no discernible discrepancies observed between the surface and the core.

To achieve a more comprehensive characterization, the microstructure was subjected to further examination using scanning electron microscopy (SEM) at higher magnifications.

**Figure 11:**
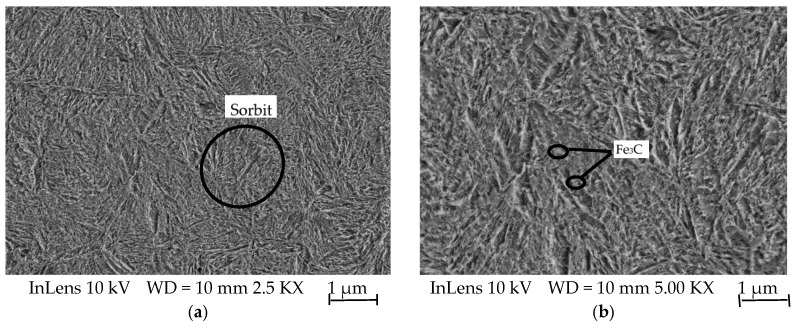
Scanning electron microscopy (SEM) microstructure of C60 steel after hardening and tempering: (**a**) core region at 2500× magnification; (**b**) core region at 5000× magnification.

As illustrated in Figure 11a, the fine lath morphology of sorbite (large circle) is indicative of a 0.6% carbon content and the application of a hardening and tempering heat treatment. At higher magnifications, Figure 11b reveals the refined distribution of ferrite and finely dispersed cementite particles, which are characteristic of a well-tempered sorbitic structure.

### 3.5. Surface Profilometry of Heat-Treated and Thermochemical Oxidation Samples

The determination of roughness for the heat-treated samples and those subjected to thermochemical oxidation was carried out by the 3D-Measurement Department of Liebherr Deggendorf, using the high-precision Zygo AMETEK device (Middlefield, CT, USA). This method facilitated a comprehensive characterization of the surfaces, meticulously accentuating the topographical alterations induced by the implemented treatments.

**Figure 12:**
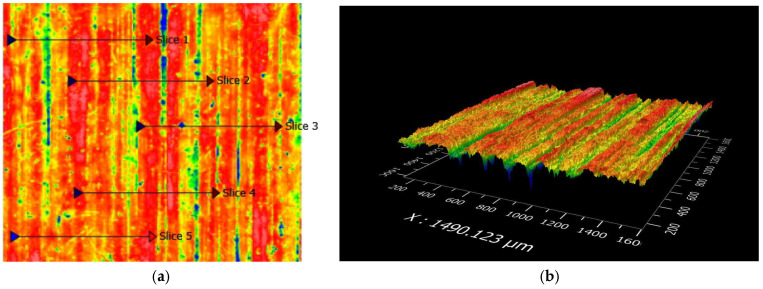
Surface profilometry of heat-treated sample: Rz = 0.794 µm: (**a**) five roughness measurements performed at distinct points across the surface; (**b**) three-dimensional topographical map of the surface.

**Figure 13:**
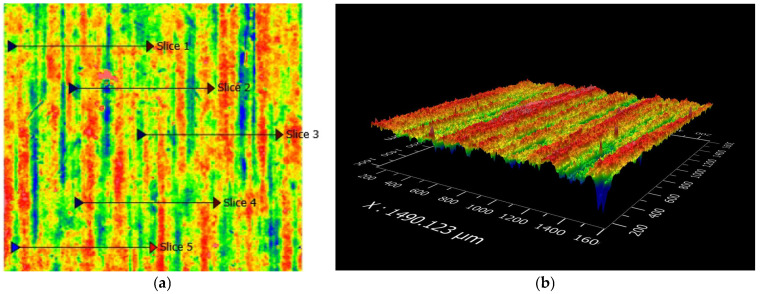
Surface profilometry of thermochemical oxidation sample: Rz = 0.861 µm: (**a**) five roughness measurements performed at distinct points across the surface; (**b**) three-dimensional topographical map of the surface.

The surface roughness of the samples was evaluated through the implementation of five measurements at varying locations on each sample (refer to Figure 12a and Figure 13a for the heat-treated and oxidized samples, respectively). In addition to the point measurements, a three-dimensional surface map was generated for each sample (see Figure 12b and Figure 13b). These maps provide a comprehensive visualization of the surface topography, highlighting the spatial distribution of roughness. The data were then subjected to statistical analysis. In this analysis, the arithmetic mean and standard deviation were calculated. These calculations ensured a robust and reproducible assessment of the treated surfaces.

The results of this study indicated a surface roughness of 0.794 µm for the heat-treated samples and 0.861 µm for the oxidized sample. While the roughness values were found to be relatively close, the hardened and tempered material exhibited slightly lower roughness, and the thermochemically oxidized material showed a marginally higher value. This increase is attributed to the formation of an oxide layer on the surface. A synopsis of the data pertaining to the roughness measurements for the two treatment types is provided in Table 4.

### 3.6. Initiation and Evolution of Fatigue Cracks in Heat-Treated Samples

**Figure 14:**
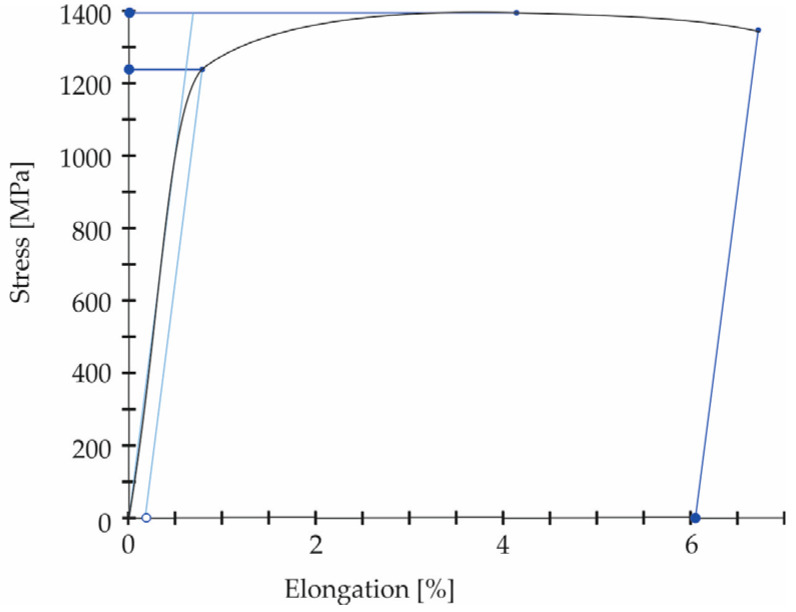
Stress–deformation diagram for hardened and tempered C60 steel.

In order to ascertain the characteristics of the heat-treated material, the stress–deformation curve was plotted in Figure 14. The elongation (A), tensile strength (Rm), and yield strength (Rp 0.2) were determined using a Zwick Typ BT1-FR250SN.A4k, 25 kN, tensile-testing machine according to DIN EN ISO 6892-1 [42].

The tensile strength of the material is presented in Table 5.

In the context of the fatigue tests conducted, the Wohler curve was not plotted; rather, a single maximum stress level was applied. This approach permitted a direct evaluation of the material’s behavior under extreme loading conditions, obviating the necessity for examining variations in durability over different stress levels.

The method was specifically selected to ascertain the initial moment of crack formation, with a focus on the triggering conditions of this phenomenon. The study’s findings indicate that by maintaining a constant stress level, the onset of material degradation can be analyzed with a high degree of precision.

The experimental protocol comprised the following steps: first, the heat-treated samples were subjected to cyclic loading in conjunction with continuous monitoring of frequency variation to identify the moment at which microcracks initiate. A total of 13 samples were subjected to rigorous testing. Initially, fatigue tests were conducted on three reference samples, which failed after a specific number of cycles (see Table 6). According to the data presented in Table 7, the average number of cycles was determined as a baseline for the subsequent testing of the remaining ten samples. The evaluation of these additional samples was conducted under conditions of progressively increasing cycles at a consistent rate. The purpose of this evaluation was to observe the formation of initial cracks.

Preliminary findings from the stress deformation test indicate that the material’s tensile strength is estimated to be approximately 1415 megapascals (MPa).

The force was determined according to the bending moment [43] and the specifications of the tested material, according to the following relation:

(1) $$F\;=\;\frac{2M}{a}\;[N]$$

where

(2) M = Rm · W [Nm]

(3) $$W={\frac{h\times \cdot b}{6}}^{2}\;({\mathrm{mm}}^{3})$$

where

- M is the bending moment,
- Rm—tensile strength,
- b = 8 mm—width of the calibrated portion,
- h = 3 mm—thickness of the sample,
- a = 20 mm—the lever arm, which refers to the distance between the points of application of forces and the points of support, and
- W—strength modulus of the cross-section (mm^3^).

Additionally, the cycles at which the machine is programmed to stop automatically are set, and frequency changes are monitored to identify any microcracks or other structural changes in the material. The number of cycles and frequency are recorded by the testing machine and subsequently tabulated. The functionality under discussion enables meticulous observation of the test parameters, thereby ensuring comprehensive documentation of the samples’ developmental trajectory throughout the experimental process.

According to the experiments, the moment of crack initiation is defined as the number of cycles at which a change in frequency is observed.

**Figure 15:**
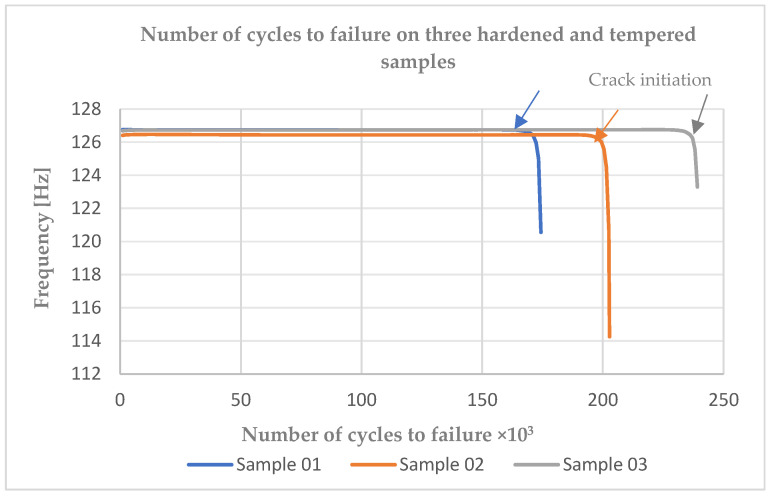
Failure diagram for hardened and tempered samples.

As illustrated in Table 8, the essential parameters, number of cycles to failure, and frequency changes for the three hardened and tempered reference samples are presented. As illustrated in Figure 15, the failure diagram reveals the frequency variation and the maximum number of stress cycles, thereby providing a schematic representation for a material that disintegrates.

This decline in frequency is indicative of material degradation resulting from repeated stress cycles. The decline in frequency indicates that the material has reached an advanced state of fatigue, characterized by a significant deterioration in its resistance to repeated loads. This deterioration approaches the material’s ultimate tensile strength, also known as the breaking point.

The following table presents the evolution of frequency until failure, highlighting the results of tests conducted on samples 01, 02, and 03. However, in all cases, the cracking of the samples occurs before this moment. A decrease in frequency has been observed to coincide with the initiation of the material’s cracking process. The table indicates that the frequency alterations observed in the three samples coincided with a decline of 0.02 Hz, indicating the onset of structural degradation.

The diagram provides a clear insight into the behavior of the material under cyclic stresses, allowing its durability under fatigue conditions to be evaluated. As illustrated by the diagram, the frequency remains constant up to a certain number of cycles at the beginning of the test. Thereafter, it commences a decrease. The decrease in frequency was identified at 163,134 cycles for sample 01, 191,177 cycles for sample 02, and 233,013 cycles for sample 03.

Table 9 presents the number of cycles until failure for each tested sample, as well as the number of cycles at which the first crack was identified. The discrepancy between these values is indicative of the material’s fatigue life subsequent to crack formation, thereby furnishing indispensable insights into its fatigue resistance and structural durability.

**Figure 16:**
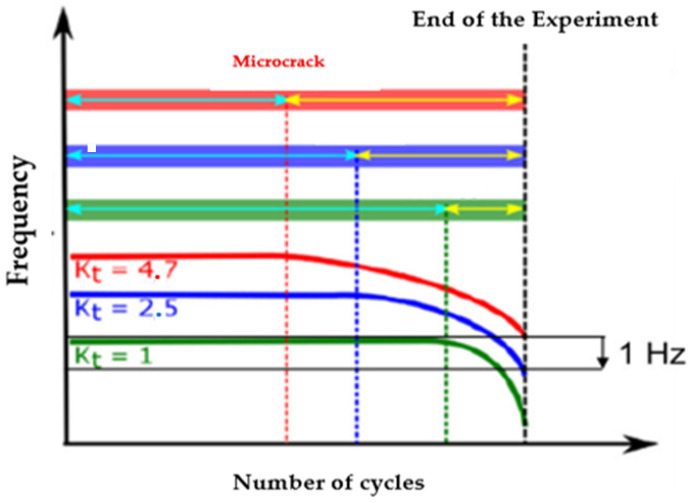
Schematic representation of the evolution of the test frequency as a function of the number of cycles.

As demonstrated in Figure 16 of Study [44], the material’s rigidity undergoes a decline once a crack emerges in the sample. This phenomenon is concomitant with a reduction in testing frequency. The blue arrow shows that the frequency remains constant, indicating that the material does not exhibit microcracks at this stage. Later, a decrease in frequency can be observed, highlighted by the yellow arrow, which confirms the appearance of microcracks and, consequently, the degradation of the material.

**Figure 17:**
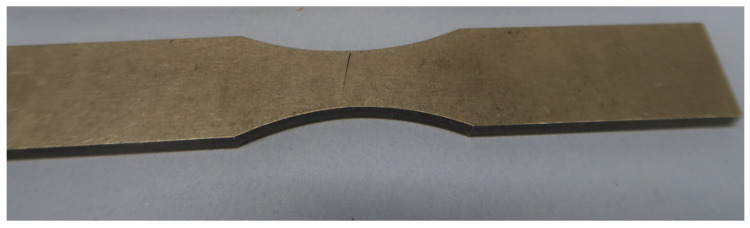
Hardened and tempered sample after fatigue test.

**Figure 18:**
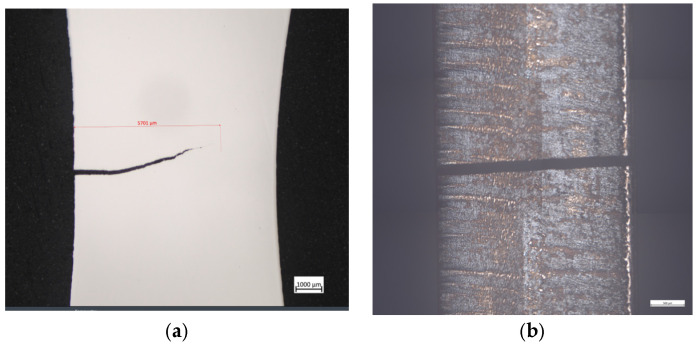
(**a**) The fatigue crack on the front side of sample 02 after 202,800 cycles [the crack length 5701 µm] and (**b**) the upper surface of the sample.

As illustrated in Figure 17, the specimen underwent a thorough testing process that led to its ultimate failure. The presence of a visible crack is evident without the necessity of conducting a CT inspection. As illustrated in Figure 18a, the frontal view of the sample following a fatigue load of 202,800 cycles is shown, while Figure 18b illustrates the upper surface of the sample. The fatigue crack was initiated in the upper region of the radius, a location where the local stress concentration typically promotes the nucleation of microcracks under cyclic loading. The length of the crack on the front surface of the sample, measured from its edge, is documented at 5701 µm. It is evident that the width of the sample in the cracked area is a mere 8 mm, thereby indicating that the length of the crack significantly exceeds half of the width of the sample.

**Figure 19:**
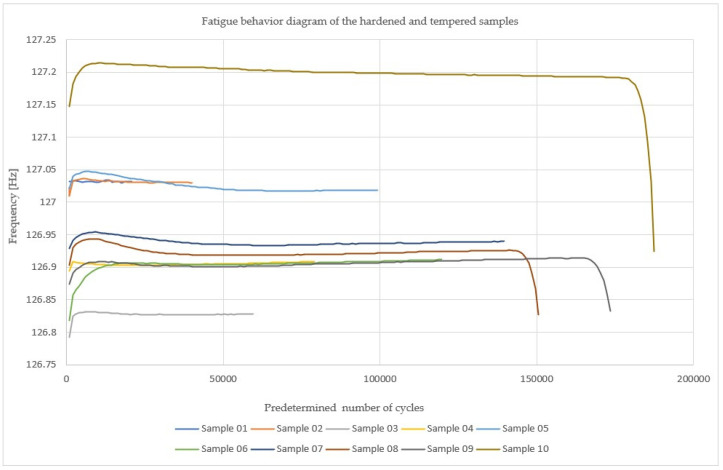
Fatigue behavior diagram for the 10 hardened and tempered samples subjected to fatigue with a predetermined number of cycles.

Table 10 presents the number of cycles established for each of the ten supplementary specimens and the frequency alterations that transpired during the experiment. Figure 19 illustrates a graph that superimposes the ten fatigue behavior curves of the hardened and tempered specimens that were subjected to a predetermined number of stress cycles. The graph presented here illustrates the variability in the fatigue behavior of the samples, thereby providing essential information for understanding how the material responds to cyclic stresses. During the testing phase, it was observed that the frequency remained constant for samples 1 through 7, exhibiting uniform behavior with minimal variation between them. However, a decrease in frequency was observed for samples 8, 9, and 10. The decrease in frequency was identified at 144,369 cycles for sample 08, 168,394 cycles for sample 09, and 174,113 cycles for sample 10.

These results indicate that the observed decrease in frequency can be considered a reliable criterion for detecting crack initiation and evaluating the fatigue behavior of the material under study. In light of the aforementioned findings, the subsequent stage of the study will entail conducting a CT analysis. This analysis will facilitate a comprehensive characterization of the cracks and their respective propagation mechanisms.

### 3.7. Computed Tomography (CT) Analysis of the Heat-Treated Material After Fatigue Testing

**Figure 20:**
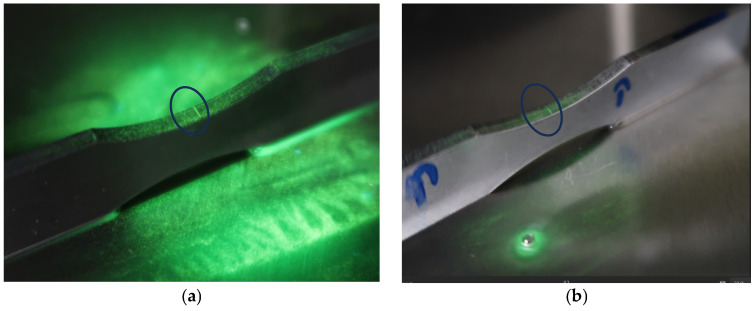

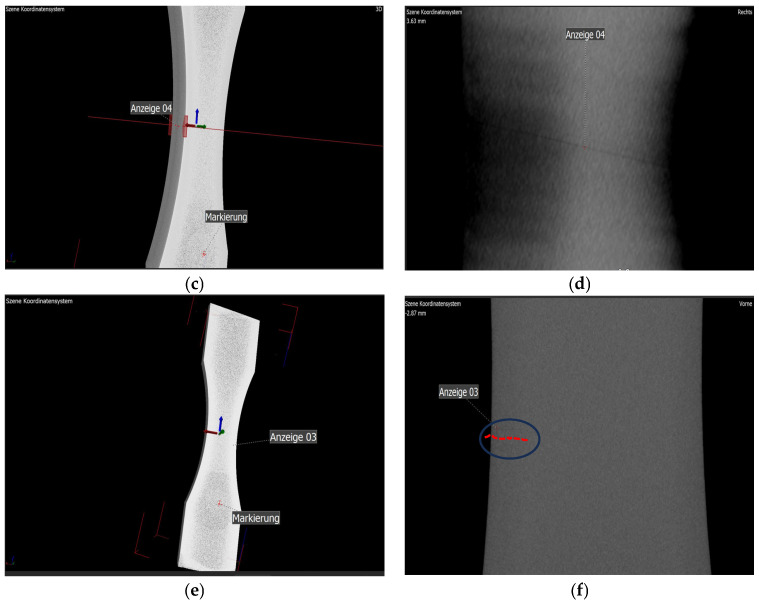
Non-destructive analysis applied to hardened and tempered sample after fatigue testing: (**a**,**b**) liquid penetrant inspection, (**c**) overview of CT examination: detail volume image—radius area, (**d**) microcrack measurement in radius area, (**e**) overview of CT examination: detail volume image—frontal surface of sample, and (**f**) microcrack measurement in the frontal surface area. The area highlighted in red represents the microcrack length, and the blue circle marks the region where the microcrack is present.

In order to verify the hypothesis that the frequency drop indicates the appearance of microcracks in the material, a computed tomography (CT) inspection was conducted. The utilization of this method enabled a comprehensive analysis of the sample’s internal structure, facilitating the confirmation of microcracks and thereby substantiating our initial hypothesis. Samples 8, 9, and 10, in which a decrease in frequency was observed, were subjected to liquid penetrant and tomographic inspection. Liquid penetrant analysis revealed the presence of a microcrack on one of the sides of the object under investigation. Conversely, computed tomography (CT) facilitated the precise identification of internal microcracks, thereby offering supplementary data regarding their dimensions and precise location within the complete volume of the material (see Figure 20). The detailed non-destructive evaluation results are presented below. As illustrated in Figure 20a,b, obtained through liquid penetrant inspection, the initial indications of surface-breaking defects in the radius area are evident. A more thorough investigation of these initial discontinuities is conducted using computed tomography. As illustrated in Figure 20c, a comprehensive overview of the CT volume in the radius region is provided, where the initiation of a microcrack is distinctly discernible. As illustrated in Figure 20d, the corresponding measurement of the microcrack indicates that it is in the initial stages of development. Figure 20e presents a CT overview of the frontal surface of the sample, where the crack has propagated from the radius toward the outer face. As illustrated in Figure 20f, the measurement of the microcrack on the frontal surface is presented. It is noteworthy that the main crack reaches a length of approximately 0.75 mm at this point. The collective analysis of the results indicates the initiation of cracks in the radius area and their subsequent propagation toward the frontal surface under conditions of decreasing fatigue frequency.

### 3.8. Initiation and Evolution of Fatigue Cracks in Oxidized Samples

**Figure 21:**
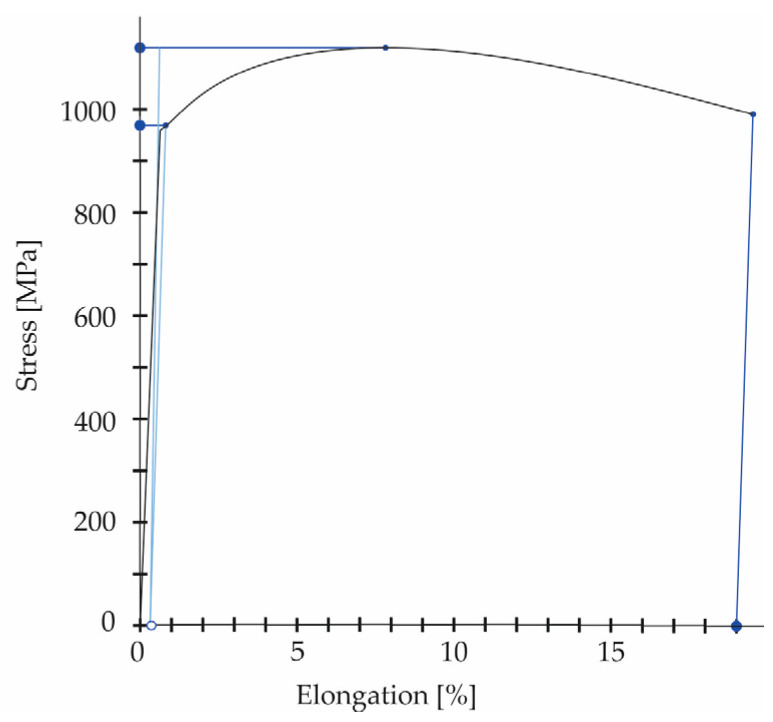
Stress–deformation diagram for oxidized C60 steel.

In order to ascertain the defining characteristics of the oxidized material, the stress–deformation curve was plotted in Figure 21. The elongation (A), tensile strength (Rm), and yield strength (Rp 0.2) were determined using a Zwick Typ UP−148525 250KN tensile-testing machine (ZwickRoell, Ulm, Germany) in accordance with DIN EN ISO 6892-1:2019 [42] The tensile strength of the material is presented in Table 11.

A discernible effect on the mechanical properties is evident following thermochemical oxidation treatment. A substantial decline in mechanical strength has been observed in the oxidized samples in comparison to the heat-treated delivery condition.

### 3.9. Fatigue Test for the Oxidized Material

The test steps were replicated for the oxidized samples in a manner consistent with the heat-treated ones. However, the machine parameters exhibited variation, a consequence of the mechanical characteristics derived from the oxidation process.

The tensile strength of the material was determined to be approximately 1119 MPa, based on the findings of the tensile test.

A total of thirteen samples were subjected to rigorous testing. The fatigue test was initially conducted on three reference samples, which failed after a specified number of cycles. In light of the resultant data, a selection of cycles was made for each of the 10 additional samples, with the objective of evaluating their behavior at varying cycle intervals.

Table 12 presents the evolution of frequency until failure, highlighting the results of tests conducted on samples 01, 02, and 03. The aforementioned tests indicated a decrease in frequency, reaching a value of 105 Hz at the moment of failure. However, in all cases, the cracking of the samples occurs before this moment. The observed decrease in frequency can be correlated with the onset of the material’s cracking process. The table indicates that the frequency exhibited changes for the three samples, concomitant with a decline of 0.02 Hz. This decline suggests the onset of structural deterioration.

**Figure 22:**
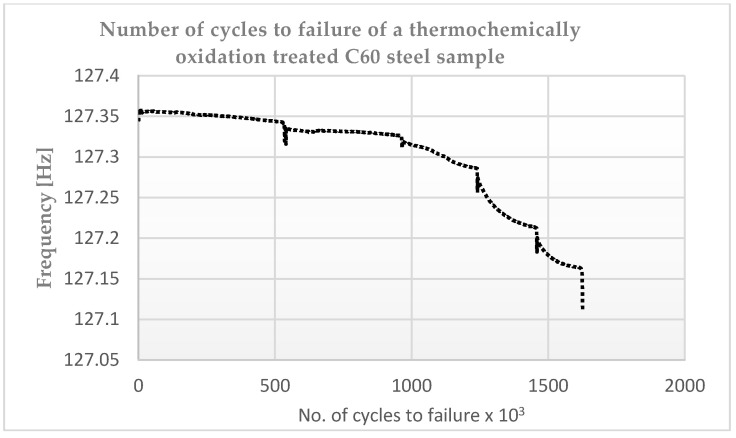
Failure diagram for thermochemically oxidation-treated sample.

As illustrated in Figure 22, the failure diagram includes both frequency variation and the maximum number of stress cycles. It has been observed that the frequency remains constant up to 540,207 cycles, indicating that the material does not show significant damage. After this point, a slight decrease in frequency begins, signaling the appearance of the first microcracks in the material. The onset of microcracks invariably leads to a reduction in the material’s stiffness. Subsequent to 965,144 cycles, the frequency persists in its decline, signifying a precipitous and imminent degradation of the material. At this stage, the microcracks have expanded significantly and have considerably affected the material structure. Ultimately, the material demonstrated complete failure after undergoing a total of 1,630,529 cycles, and the final recorded frequency exhibited a notably low value.

**Figure 23:**
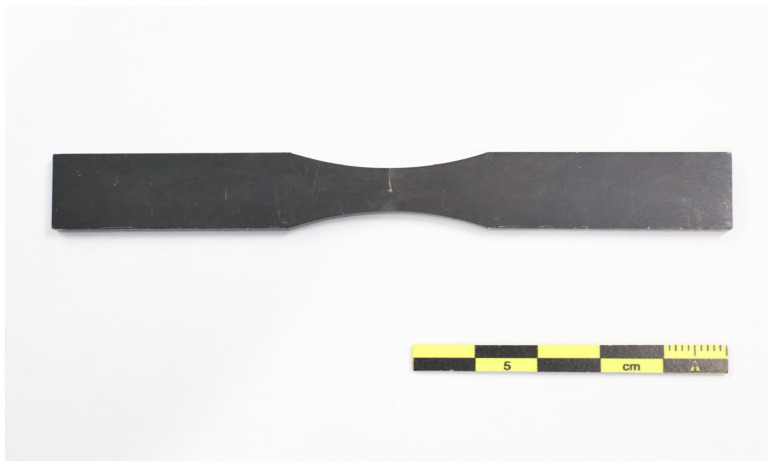
Oxidized sample after the breaking test.

**Figure 24:**
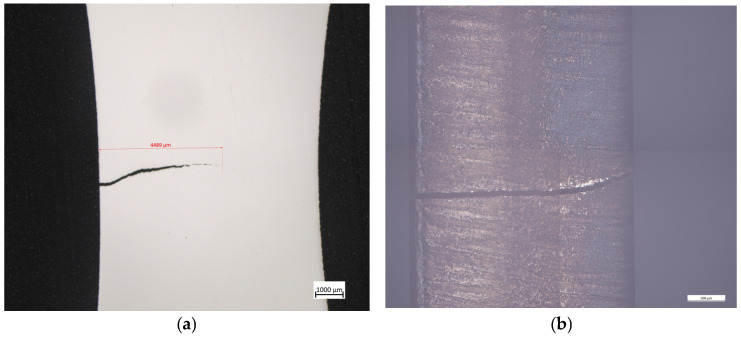
(**a**) The fatigue crack on the front side of sample 01 after 1,630,529 cycles, and (**b**) the upper surface of the sample: the crack length is 4489 µm.

Table 13 presents the number of cycles until failure for each tested sample, as well as the number of cycles at which the first crack was identified. The discrepancy between these values is indicative of the material’s fatigue life subsequent to crack formation, thereby furnishing indispensable insights into its fatigue resistance and structural durability:

As illustrated in Figure 23, the oxidized sample was subjected to testing until failure. As with the hardened and tempered specimen, a visible crack is evident without the necessity of conducting a CT inspection. As illustrated in Figure 24a, the frontal view of the sample following a fatigue load of 1,630,529 cycles is shown. Figure 24b illustrates the upper surface of the sample.

Fatigue crack initiation occurred in the upper region of the radius, where the stress field is locally intensified under cyclic loading. Its progression is characterized by a linear trajectory with minimal branching or deviation. The crack length on the frontal surface of the specimen, measured from its edge, is documented at 4489 µm, corresponding to approximately one-third of the specimen’s total width.

**Figure 25:**
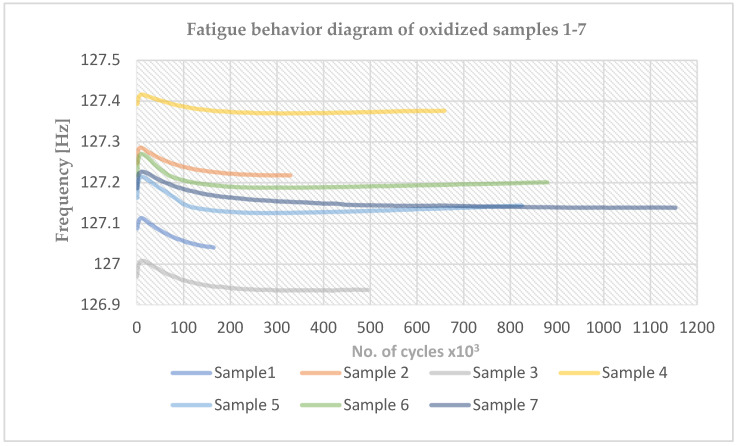
Fatigue behavior diagram of samples 1–7 thermochemically treated by oxidation and subjected to fatigue with a predetermined number of cycles.

**Figure 26:**
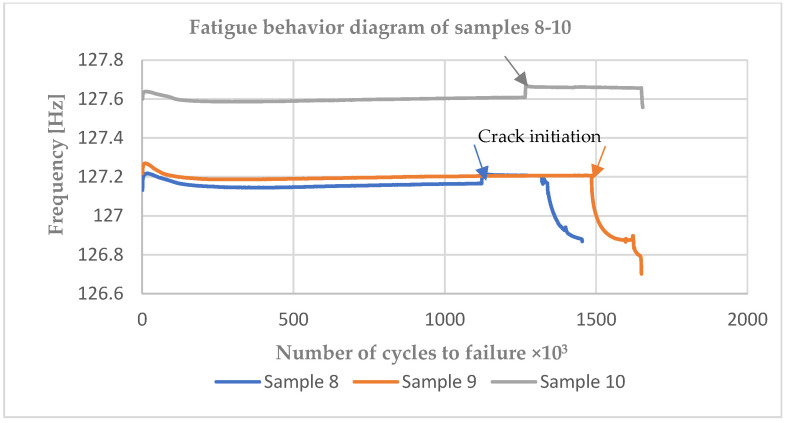
Fatigue behavior diagram of samples 8–10 thermochemically treated by oxidation and subjected to fatigue with a predetermined number of cycles.

As illustrated in Table 14, the number of cycles set for each sample of the 10 additional samples is documented, along with the frequency changes that occurred during the experiment. Figure 25 presents a schematic representation of the superposition of seven curves, illustrating the fatigue behavior of samples subjected to thermochemical oxidation, which exhibited no frequency changes. Figure 26 demonstrates samples 8, 9, and 10, for which a substantial decrease in frequency was observed. A decrease in frequency, with a mean value of 0.6 Hz, was observed for these samples.

No frequency change was observed during the testing of samples 1 through 7. This finding suggests that the material exhibited no microcracking, thereby demonstrating uniform strength and stable structural integrity under cyclic stresses.

Samples 8, 9, and 10 demonstrate that, following the development of microcracks, the material exhibits resilience in resisting cyclic loading.

During the testing phase, it was observed that the frequency remained constant for samples 1 through 7, exhibiting uniform behavior with minimal variation between them. However, a decrease in frequency was observed for samples 8, 9, and 10. The decrease in frequency was identified at 1,084,258 cycles for sample 08, 1,482,668 cycles for sample 09, and 1,263,405 cycles for sample 10.

### 3.10. Computed Tomography (CT) Analysis of the Oxidized Sample After Fatigue Testing

In order to verify the hypothesis that the frequency drop indicates the appearance of microcracks in the material, a computed tomography (CT) inspection was conducted. The utilization of this method enabled a comprehensive analysis of the sample’s internal structure, facilitating the confirmation of microcracks and thereby substantiating our initial hypothesis.

**Figure 27:**
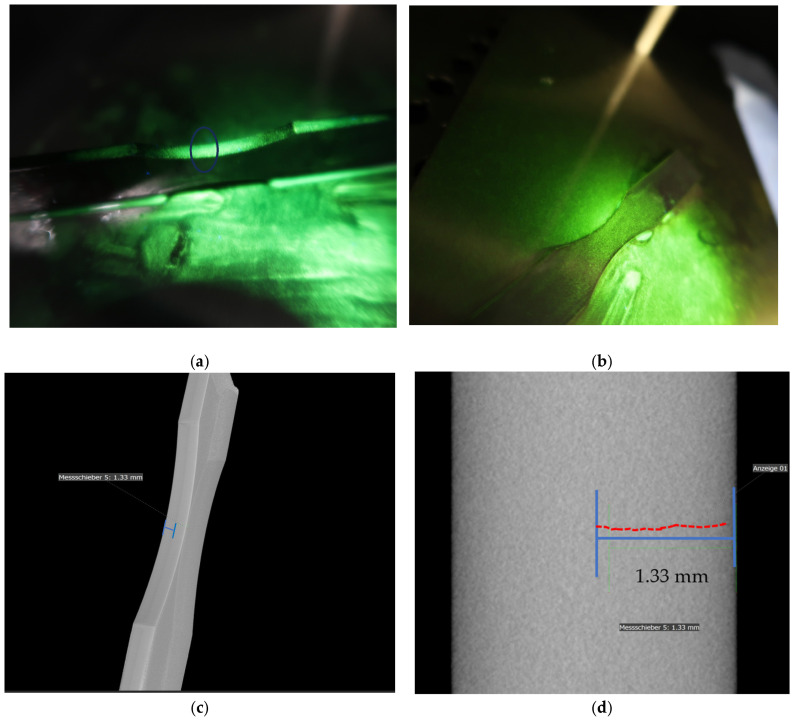

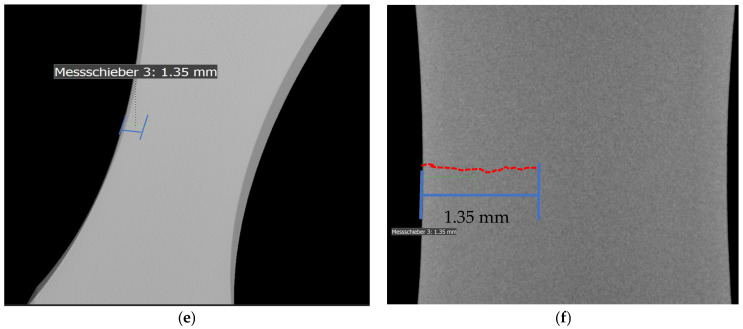
Non-destructive analysis applied to oxidized sample after fatigue testing: (**a**,**b**) liquid penetrant inspection, and (**c**) overview of CT examination: detail volume image—radius area, (**d**) microcrack measurement in radius area, (**e**) overview of CT examination: detail volume image-frontal surface of sample, and (**f**) microcrack measurement in the frontal surface area. The red line indicates the microcrack length, while the blue line represents the measured value obtained during the analysis.

Samples 8–10, in which a decrease in frequency was observed, were subjected to liquid penetrant and tomographic inspection. Liquid penetrant analysis revealed the presence of a microcrack on one of the sides. Conversely, computed tomography (CT) facilitated the precise identification of internal microcracks, thereby offering supplementary data regarding their dimensions and precise location within the material’s complete volume (see Figure 27).

The specimen initially reveals a fracture in the radius region, which subsequently propagates to the frontal surface. The crack size in the radius area is 1.33 mm, while on the frontal surface, the crack measures 1.35 mm. This evolution of the crack suggests a propagation of the defect from the radial area to the frontal surface. Consequently, the critical points of the material are emphasized.

## 4. Fracture Surface Examination

A fracture surface examination was performed to identify the crack initiation sites and the dominant fracture mechanisms. A detailed SEM fractographic analysis was carried out on the fractured samples, including both the heat-treated and the oxidized specimens, in order to evaluate their fatigue behavior. The SEM investigation enabled high-resolution documentation of the fracture surfaces, revealing the characteristic crack paths and morphological features associated with fatigue failure. The fracture path is particularly evident in the upper region of the fracture surface, where the crack initiation zone is located. The primary fracture zone is characterized by a preponderance of cleavage planes, which are hallmarks of fatigue fracture in this alloy. These planes suggest a brittle-like propagation mechanism under cyclic loading conditions.

**Figure 28:**
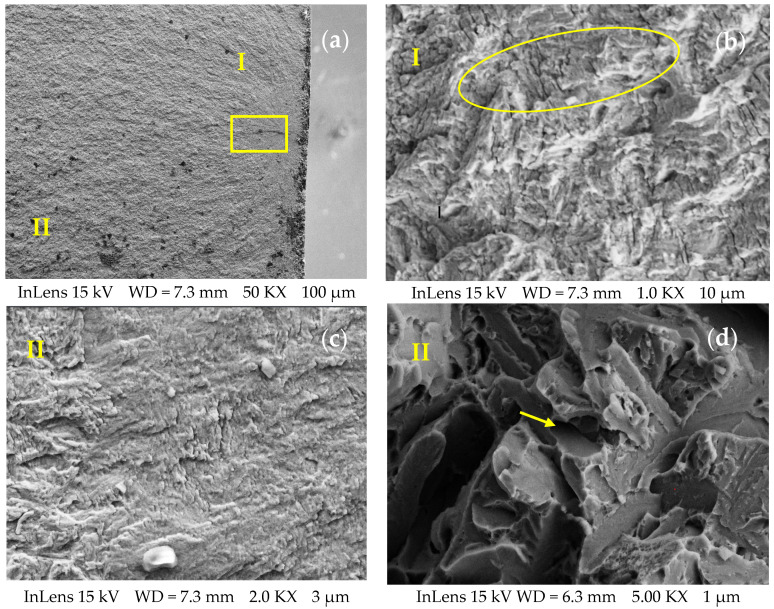
(**a**) Fatigue fracture surface of the hardened and tempered sample; (**b**–**d**) detailed views of the different fracture areas.

As illustrated in Figure 28a, the images demonstrate that crack initiation, demarcated by the rectangular marker, invariably occurs within the transition radius. This region is distinguished by elevated stress concentration under cyclic loading conditions.

Additionally, two distinct fracture areas are observable in the specimen (see Figure 28b–d). In the fracture zone I, the presence of a surface morphology indicative of fatigue failure is evident. This morphology is characterized by the presence of well-defined arrest lines, which are oriented perpendicularly to the crack propagation direction. These characteristics are indicative of the progressive, stage-wise advancement of the crack under cyclical loading. In contrast, fracture zone II demonstrates the hallmark characteristics of overload failure, manifesting as a ductile honeycomb morphology that materializes through the coalescence of microvoids during the final stage of fracture.

**Figure 29:**
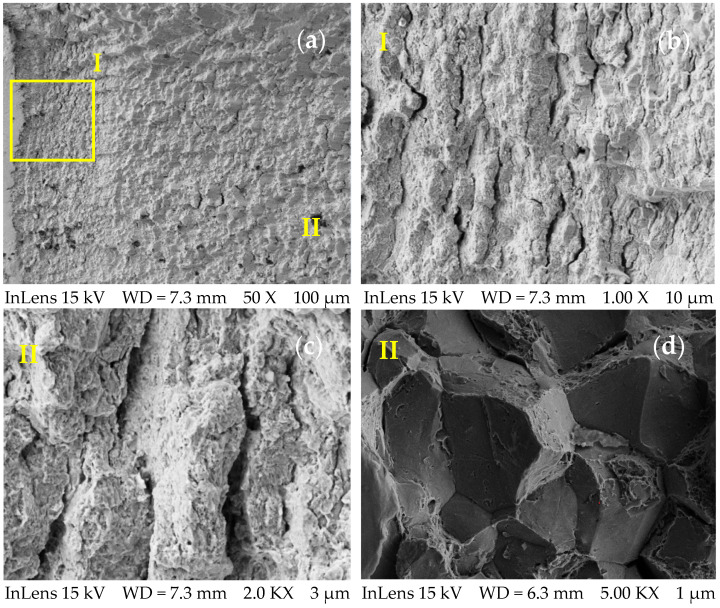
(**a**) Fatigue fracture surface of oxidized sample; (**b**–**d**) detailed views of the different fracture areas.

As illustrated in Figure 29a–d, the fracture surface of the oxidized samples is presented at various magnifications. As illustrated in Figure 29a,b, the fracture surface demonstrates a relatively uniform morphology, characterized by a smooth crack path and an absence of substantial microcrack development. It has been established that these regions correspond to the initial stages of crack propagation. During this stage, the oxide layer remains continuous and effectively limits the opening of superficial discontinuities. Figure 29c,d, captured at higher magnifications, reveal localized areas where microcracks begin to form within the oxide layer.

As illustrated in Figure 27f and Figure 29d, the oxidized samples exhibit a higher density of microcracks. Nevertheless, the oxide layer mitigates local stress concentrations and contributes to the reduction in the propagation velocity of the primary crack. Furthermore, the oxide scale functions as a rigid superficial barrier, thereby ensuring more uniform stress distribution and restricting the opening of individual microcracks.

While microcracks are more prevalent, they tend to be superficial and do not typically progress into dominant cracks, thereby slowing the propagation of the critical crack. While the oxidation treatment results in a reduction of tensile strength, it generates a stable surface layer that modifies the stress distribution at the surface and introduces compressive residual stresses. The initiation of fatigue cracks is delayed by these compressive stresses which also reduce the influence of local maximum stress during the early stages of cyclic loading. Furthermore, the oxide layer has been demonstrated to impede the propagation of incipient cracks, thereby mitigating their potential to cause damage to the material.

## 5. Statistical Analysis

A rigorous statistical analysis was conducted to quantitatively assess the disparities between the heat-treated and oxidized samples. This analysis was undertaken to ascertain the scientific relevance of the experimentally observed trends. The objective evaluation of the contribution of each stage to the total fatigue life is facilitated by expressing the fatigue life before and after microcrack initiation in percentage terms. This approach reduces the influence of sample-to-sample variability and strengthens the robustness of interpretation. This approach provides a solid analytical framework for understanding how heat treatment and oxidation influence both crack nucleation and the subsequent propagation mechanisms.

**Figure 30:**
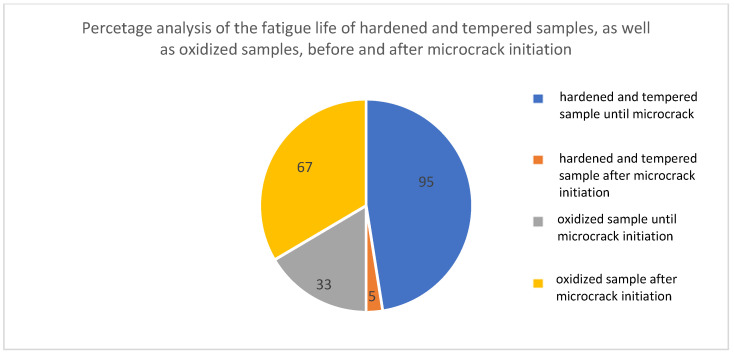
Percentage analysis of the fatigue life of hardened and tempered samples, as well as oxidized samples, before and after microcrack initiation.

As illustrated in Figure 30, there are substantial discrepancies between heat-treated and oxidized samples with regard to the distribution of fatigue life. The samples that underwent heat treatment exhibited a predominant proportion of their fatigue life prior to microcrack initiation (95%), suggesting a high degree of resistance to crack nucleation. Conversely, the oxidized samples exhibited a significantly reduced proportion of this stage (33%), indicating an augmented vulnerability to crack initiation. Furthermore, in the oxidized samples, a significant portion of fatigue life is utilized during the crack propagation stage (67%), thereby reflecting the impact of the oxide layer on the mechanisms governing crack growth. Therefore, the results provide a clear comparative perspective on how heat treatment and oxidation affect both crack initiation and crack evolution. This, in turn, reinforces the conclusions regarding the fatigue behavior of the material. The findings indicate that while oxidized samples exhibit enhanced longevity, they also manifest a propensity for accelerated crack formation in comparison to heat-treated samples.

## 6. Conclusions

- This study offers direct comparison between quenched-and-tempered and thermochemically oxidized C60 steel, with both specimens subjected to identical four-point bending fatigue conditions.
- Thermochemical oxidation has been demonstrated to enhance fatigue life by approximately eightfold, from 205,700 cycles for quenched-and-tempered samples to 1,628,681 cycles for oxidized samples.
- A salient finding of the study is that oxidized samples exhibit an accelerated initiation of microcracks, yet their propagation is markedly retarded due to the protective oxide layer and the compressive residual stress that is generated during the oxidation process.
- In contrast, quenched-and-tempered samples exhibit a delayed initiation of cracks; however, once these cracks form, they propagate rapidly, thereby reducing the overall fatigue performance.
- The present study sought to ascertain the correlation between microstructure and mechanical behavior. To this end, a variety of analytical techniques were employed, including GDOES, SEM, CT scanning, and frequency monitoring. These methods were utilized to confirm the uniformity of the oxide layer and to determine its beneficial role in delaying crack growth.
- Frequency-based monitoring has been demonstrated to serve as a reliable early indicator of microcrack initiation, thereby validating its application in high-frequency fatigue testing.
- Overall, thermochemical oxidation is the preferred method for applications that necessitate an extended fatigue life. However, it is important to note that the optimization of treatment parameters and microstructural characterization can further enhance material reliability under cyclic loading.

It is imperative to note that the findings of this study are exclusively applicable to C60 steel, given the profound influence of microstructure and hardness on fatigue behavior. The generalization of these findings to other medium-carbon steels would necessitate further research.

## Figures and Tables

**Table 1:** Chemical composition of C60 [1.0601] material purchased in heat-treated condition by hardening and tempering.

Alloy Composition (at. %)	C	Si	Mn	P	S	Cr	Mo	Ni	Cr + Mo + Ni
DIN EN ISO 683-1:2018 [40]	0.57–0.65	max. 0.4	0.60–0.90	max. 0.045	max. 0.045	max. 0.40	max. 0.10	max. 0.40	max. 0.63
Analyzed sample	0.64	0.22	0.61	0.001	0.004	0.22	0.006	0.022	0.248

**Table 2:** Hardness measurements on samples thermochemically treated by oxidation.

Number of Measurements	Microhardness of the Sample Surface	Mechanical Strength	Hardness of the Core	Mechanical Strength
1	440 HV 0.05	1420 MPa	293 HV 30	918 MPa
2	455 HV 0.05	1455 MPa	293 HV 30	918 MPa
3	460 HV 0.05	1485 MPa	295 HV 30	924 MPa

**Table 3:** Hardness measurements on the sample as hardened and tempered.

Number of Measurements	Hardness of the Sample Surface	Mechanical Strength	Hardness of the Core	Mechanical Strength
1	416 HBW	1317 MPa	423 HV 30	1320 MPa
2	414 HBW	1310 MPa	424 HV 30	1323 MPa
3	414 HBW	1310 MPa	424 HV 30	1323 MPa

**Table 4:** The results of roughness measurements for heat-treated and oxidized samples.

Number of Measurements	Rz [µm]	
	Heat-Treated Sample	Oxidized Sample
1	0.843	0.626
2	0.868	0.821
3	0.982	1.229
4	0.734	0.807
5	0.545	0.824
	$\bar{x}$ = 0.794	$\bar{x}$ = 0.861

**Table 5:** The characteristics of the heat-treated material.

Sample	a_0_ [mm]	b_0_ [mm]	S_0_ [mm^2^]	L_e_ [mm]	m_E_ [GPa]	R_p0.2_ [MPa]	F_m_ [kN]	R_m_ [MPa]	A_10mm_ [%]
01	3.04	8.02	24.38	10.01	206	1257	34.49	1415	6.0

**Table 6:** Number of cycles to failure.

Sample 01	174,600	$\bar{x}$ = 205,700 [samples tested to failure]
Sample 02	202,800	
Sample 03	239,700	

**Table 7:** Samples tested for fatigue at a predetermined number of cycles.

Sample 01	20,000	Samples tested for fatigue at a predetermined number of cycles
Sample 02	40,000	
Sample 03	60,000	
Sample 04	80,000	
Sample 05	100,000	
Sample 06	120,000	
Sample 07	140,000	
Sample 08	160,000	
Sample 09	180,000	
Sample 10	200,000	

**Table 8:** Fatigue to failure test results for the hardened and tempered samples.

No. of Sample	Total No. of Cycles	Initial Frequency [Hz]	Decrease in Frequency [Hz]	Final Frequency [Hz]
01	174,600	126.760	126.736	120.558
02	202,800	126.442	126.437	114.239
03	239,700	126.709	126.674	123.297
	$\bar{x}$ = 205,700	$\bar{x}$ = 126.637	$\bar{x}$ = 126.615	$\bar{x}$ = 119.364

**Table 9:** Analysis of the hardened and tempered samples’ fatigue life after crack formation.

No. of Sample	Cycles at Crack Initiation	Cycles Until Failure	Remaining Fatigue Life After Crack Initiation (Cycles)
01	163,134	174,600	14,466
02	191,177	202,800	11,623
03	233,013	239,700	6687
	$\bar{x}$ = 195,774	$\bar{x}$ = 205,700	$\bar{x}$ = 10,925

**Table 10:** Results obtained after fatigue testing with a predetermined number of cycles for the hardened and tempered samples.

No. of Sample	No. of Cycles	Initial Frequency [Hz]	Final Frequency [Hz]
1	20,000	127.032	127.032
2	40,000	127.034	127.034
3	60,000	126.824	126.824
4	80,000	126.908	126.908
5	100,000	127.021	127.021
6	120,000	126.900	126.900
7	140,000	126.929	126.928
8	160,000	126.930	126.826
9	180,000	126.900	126.832
10	200,000	127.200	126.924

**Table 11:** The characteristics of the heat-treated material the thermochemical oxidation.

Sample	a_0_ [mm]	b_0_ [mm]	S_0_ [mm^2^]	L_e_ [mm]	m_E_ [GPa]	R_p0.2_ [MPa]	F_m_ [kN]	R_m_ [MPa]	A_10mm_ [%]
01	3.05	7.99	24.37	10.05	217	970	27.27	1119	18.8

**Table 12:** Fatigue to failure test results for oxidized samples.

No. of Sample	No. of Cycles	Initial Frequency [Hz]	Decrease in Frequency [Hz]	Final Frequency [Hz]
01	1,630,529	127.352	127.330	105.208
02	1,627,490	127.350	127.342	105.192
03	1,628,025	127.368	127.340	105.189
	$\bar{x}$ = 1,628,681	$\bar{x}$ = 127.35	$\bar{x}$ = 127.33	$\bar{x}$ = 105.19

**Table 13:** Analysis of the oxidized samples’ fatigue life after crack formation.

No. of Sample	Cycles at Crack Initiation	Cycles Until Failure	Fatigue Life After Crack Initiation (Cycles)
01	540,207	1,630,529	1,090,322
02	540,352	1,627,490	1,087,138
03	539,980	1,628,025	1,088,048
	$\bar{x}$ = 540,179	$\bar{x}$ = 1,628,681	$\bar{x}$ = 1,088,502

**Table 14:** Results obtained after fatigue testing with a predetermined number of cycles for the oxidized sample.

No. of Sample	No. of Cycles	Initial Frequency [Hz]	Final Frequency [Hz]
1	165,000	127.08	127.08
2	330,000	127.24	127.24
3	495,000	126.97	126.97
4	660,000	127.39	127.39
5	825,000	127.18	127.18
6	990,000	127.19	127.19
7	1,155,000	127.18	127.18
8	1,320,000	127.20	126.86
9	1,485,000	127.21	126.70
10	1,650,000	127.60	126.50

## Data Availability

The original contributions presented in this study are included in the article. Further inquiries can be directed to the corresponding author.

## Appendix A

**Table A1:** The planning of the experiment.

No.	Stage	Targeted Results	Type of Testing	Place of Measurement
1.	Procurement of materials for experiments	110 × 14 × 3 mm		Fa. Zelos Zerspanung Inh. Simon Oreskovic e. K.- Bessenbach, Germany
2.	Check the chemical composition of the material	Composition of the material	Optical emission spectrometer	Fa. Liebherr Deggendorf
3.	Identification of samples	Laser marking	Laser	Fa. Liebherr Deggendorf
4.	Thermochemical treatment by oxidation	Gas oxidation	Gas furnace	Fa. Härterei Reese Brackenheim
5.	Analysis of hardness and microhardness	Obtained results	Microhardness tester	Fa. Liebherr Deggendorf
6.	Metallography analyses	Determination of microstructure	Stereo- and light-microscopy	Fa. Liebherr Deggendorf
7.	Fatigue testing	Number of cycles to fracture	Rumul MIKROTRON, a resonant testing machine for fatigue tests up to 20 kN	Fa. Liebherr Deggendorf
8.	Non-destructive testing of samples	Crack identification with magnetic powder	NDT-MT-testing [magnetic particle testing)	Fa. Liebherr Deggendorf
		Crack length measurement by computer tomography	CT (Computer tomography)	Technische Hochschule Deggendorf
		Fracture surface examination	Scanning Electron Microscopy (SEM) analysis	Technische Hochschule Deggendorf
9.	The characterization of the oxide layer	Analyzing the composition and thickness of the oxide layer formed on a material’s surface	GDOES (Glow Discharge Optical Emission Spectroscopy)	TAZ GmbH
